# Parameter-Based Evaluation of Attentional Impairments in Schizophrenia and Their Modulation by Prefrontal Transcranial Direct Current Stimulation

**DOI:** 10.3389/fpsyt.2017.00259

**Published:** 2017-11-29

**Authors:** Nadine Gögler, Irina Papazova, Tatiana Oviedo-Salcedo, Nina Filipova, Wolfgang Strube, Johanna Funk, Hermann J. Müller, Kathrin Finke, Alkomiet Hasan

**Affiliations:** ^1^Department of Psychology, Ludwig-Maximilians-Universität München, München, Germany; ^2^Department of Psychiatry and Psychotherapy, Ludwig-Maximilians-Universität München, München, Germany; ^3^Hans-Berger-Department of Neurology, Friedrich-Schiller-Universität Jena, Jena, Germany

**Keywords:** transcranial direct current stimulation, schizophrenia, visual processing speed, visual short-term memory, theory of visual attention, dorsolateral prefrontal cortex

## Abstract

**Background:**

Attentional dysfunctions constitute core cognitive symptoms in schizophrenia, but the precise underlying neurocognitive mechanisms remain to be elucidated.

**Methods:**

In this randomized, double-blind, sham-controlled study, we applied, for the first time, a theoretically grounded modeling approach based on Bundesen’s Theory of Visual Attention (TVA) to (i) identify specific visual attentional parameters affected in schizophrenia and (ii) assess, as a proof of concept, the potential of single-dose anodal transcranial direct current stimulation (tDCS; 20 min, 2 mA) to the left dorsolateral prefrontal cortex to modulate these attentional parameters. To that end, attentional parameters were measured before (baseline), immediately after, and 24 h after the tDCS intervention in 20 schizophrenia patients and 20 healthy controls.

**Results:**

At baseline, analyses revealed significantly reduced visual processing speed and visual short-term memory storage capacity in schizophrenia. A significant stimulation condition × time point interaction in the schizophrenia patient group indicated improved processing speed at the follow-up session only in the sham condition (a practice effect), whereas performance remained stable across the three time points in patients receiving verum stimulation. In healthy controls, anodal tDCS did not result in a significant change in attentional performance.

**Conclusion:**

With regard to question (i) above, these findings are indicative of a processing speed and short-term memory deficit as primary sources of attentional deficits in schizophrenia. With regard to question (ii), the efficacy of single-dose anodal tDCS for improving (speed aspects of visual) cognition, it appears that prefrontal tDCS (at the settings used in the present study), rather than ameliorating the processing speed deficit in schizophrenia, actually may interfere with practice-dependent improvements in the rate of visual information uptake. Such potentially unexpected effects of tDCS ought to be taken into consideration when discussing its applicability in psychiatric populations. The study was registered at http://apps.who.int/trialsearch/Trial2.aspx?TrialID=DRKS00011665.

## Introduction

Visual attention dysfunctions, ranging from impairments in processing speed and visual short-term memory (vSTM) capacity to deficient top-down control (1–6), are commonly reported in schizophrenia and schizophrenia-spectrum disorders. However, the question of the precise neurocognitive mechanisms underlying the difficulties in attention tasks has not yet been resolved conclusively. For instance, it remains elusive whether both processing speed and working memory (WM) functions are affected in schizophrenia (7) or whether slowed encoding processes are responsible for the reduced vSTM storage capacity in the respective attention tasks (2, 8, 9). Likewise, it is not clear whether the impaired encoding processes arise from impaired top-down controlled distractor inhibition (10–14).

To determine whether these deficits can be attributed to losses of specific fundamental attention functions, a theoretically grounded modeling approach is required that can isolate and quantify (potentially compromised) core functions in an unconfounded measurement. Such an approach is provided by Bundesen’s Theory of Visual Attention [TVA; (15)], which already proved valuable for systematically characterizing cognitive deficits in diverse neuropsychiatric and neurological disorders (16–18). By combining this framework theory with simple psychophysical tests of whole- and partial-report of briefly presented letters, it is possible to derive independent estimates of parameters reflecting the individual efficiency of core visual attention functions. Two of these parameters, visual processing speed, the rate of information uptake per second (*C*), and vSTM storage capacity, the maximum number of visual objects that can be represented at one time (*K*), capture general capacity aspects of the system; and the top-down control parameter (α) describes the system’s (top-down) attentional selectivity. The ability of the TVA-based approach to provide “process-pure” and independent measures of the various attention functions has been demonstrated in a range of studies revealing disorder-specific patterns of attentional deficits, for instance, selective impairment in only one parameter but not the others (16, 17). Similarly, in healthy individuals, externally induced modulations of the alertness level have been shown to specifically increase processing speed, without influencing vSTM storage capacity (19). Furthermore, as the tasks do not require speeded responses, the parameters can be estimated uninfluenced by (e.g., antipsychotic drug-induced) motor side effects. Importantly also, unlike most standard neurocognitive tests, TVA-based assessment is highly sensitive so that even subtle deviations of cognitive performance from the norm can reliably be detected (20). Given these advantages, the TVA-based approach is well suited for the prime purpose of the present study: to identify the specific attentional functions that are compromised in schizophrenia.

A secondary aim of this study was to investigate whether the compromised attentional performance in schizophrenia patients can be modulated by means of prefrontal transcranial direct current stimulation (tDCS). On a neuronal level, abnormal activation patterns within dorsolateral prefrontal cortex (dlPFC) attention networks are discussed as the underlying source of these attentional impairments (13, 21–25). Accordingly, modulation of intrinsic prefrontal networks through tDCS has recently been proposed as potential non-invasive and safe treatment option for the remediation of cognitive dysfunctions in schizophrenia patients (26, 27). tDCS modulates cortical excitability by passing small direct currents on to the scalp *via* electrodes with anodal and cathodal polarity. While short-term tDCS effects are attributed to tonic modulations of the resting membrane potential of cortical neurons affecting their firing rates, prolonged after-effects are presumed to be controlled by protein synthesis-dependent processes at the synaptic level (28–31). Preliminary studies already provided promising results regarding the potential of tDCS to remediate cognitive deficits in psychiatric diseases, for example, in patients with major depression (18, 32, 33) or alcohol dependence (34). However, with respect to schizophrenia, the available evidence is scarce and mixed (35, 36): one study applied 20 min of anodal tDCS with 2 mA to the left dlPFC and could not show that anodal tDCS improves performance on a procedural learning task in the whole sample, but still had a beneficial effect in a subgroup of patients (37). Another single-session experiment reported a positive effect of 2 mA anodal, but not 1 mA or sham, tDCS to the left dlPFC on a WM task, 20 and 40 min after stimulation (38). By contrast, in another study, a similar stimulation protocol was shown to be ineffective to influence cognitive functions measured by the MATRICS consensus cognitive battery composite score (39). To expand our knowledge about the possible efficacy of tDCS in schizophrenia, in the second step of this proof-of-principle study, we explored whether the modulation of intrinsic networks through single-dose tDCS can have a functional significance for cognitive, and more specifically, visual attentional processes in schizophrenia (40). As anodal tDCS applied to the left dlPFC was previously shown to modulate intrinsic fronto-parietal networks in healthy humans, the beneficial cognitive effect of prefrontal tDCS has been attributed to an increase of the state of alertness (41). Consequently, we hypothesized that prefrontal tDCS would influence particularly alertness-dependent cognitive processes, such as the speed by which visual stimuli are processed (42–44). On the other hand, tDCS could also affect other attentional components such as vSTM storage capacity or attentional selectivity, subserved, at least partly, by prefrontal cortex and its functional and structural connections.

Measures assessing tDCS-induced benefits should be able to disentangle the potential effects on different attentional component processes subserved by prefrontal cortex (13, 38, 45, 46). Furthermore, as the effects induced by single-dose tDCS are subtle (47, 48), highly sensitive tools are a prerequisite for reliably detecting any (likely small) modulations of the various cognitive sub-processes. Previous studies using pharmacological interventions or cue stimuli have already revealed the high sensitivity of TVA parameters even to small manipulations of the alertness level (19, 42–44). In this respect, TVA-based parametric attentional assessment provides, arguably, the best available tool for the aims of the present study, to (i) create a meaningful “attentional profile” of schizophrenia patients and (ii) to examine for (subtle) tDCS-induced changes in attentional functions in these patients.

## Methods

### Participants

20 patients with a ICD-10 diagnosis of schizophrenia or schizophrenia-spectrum disorder (F20 = 19; F25 = 1), recruited from the Department of Psychiatry and Psychotherapy (LMU Munich), and the same 20, demographically matched, healthy controls that participated in our previous study (18), were included in the investigation (see Tables 1 and 2 for demographic and clinical data). The diagnoses, according to the WHO ICD-10 criteria for schizophrenia or schizophrenia-spectrum disorder, were made by two clinical psychiatrists of whom one (Alkomiet Hasan) is a member of this study group. The period of recruitment lasted from May 2015 until October 2016. The trial ended after the target sample size was reached. The sample size was estimated from previous experimental studies investigating the effect of alertness manipulations on TVA parameters in healthy participants (42, 43). Patients were assessed for psychopathological symptoms [Positive and Negative Syndrome Scale (PANSS); Calgary Depression Rating Scale for Schizophrenia (CDSS)] (49, 50), disease severity [Clinical Global Impression Scale (CGI)] (51), and functioning [Global Assessment of Functioning Scale (GAF)] (52). The clinical rater (Irina Papazova) was not involved in any other aspects of the study and had undergone extensive training in the use of the scales. Participants with a contraindication to tDCS were excluded. Further exclusion criteria were an IQ below 86 (German Multiple-Choice Vocabulary Test MWT-B) (53), red–green color blindness, and suicidal intent. All except one patient received second-generation antipsychotics and one patient received an additional first-generation antipsychotic medication. 68% of the patients received antipsychotic monotherapy. Furthermore, all patients were clinically stable as indicated by the PANSS values (see Table 2).

**Table 1:** Group demographics.

	Schizophrenia patients	Healthy controls	*p*-Value
Age	36.55 (9.16)	31.7 (8.31)	0.09
Gender (m/f)	13/7	10/10	0.34
Handedness (r/l/a)	18/1/1	18/2/0	0.51
Education (years)	10.5 (1.57)	12.8 (0.37)	0.01
Verbal IQ (MWT-B)	106.88 (16.11)	112.2 (18.64)	0.37

*Data are presented as means ± SDs or frequencies*.

*MWT-B, German Multiple-Choice Vocabulary Test; f, female; m, male; r, right; l, left; a, ambidextrous*.

*p-Values refer to a statistical comparison between the schizophrenia patient and healthy control group*.

**Table 2:** Comparison of demographics and clinical ratings for verum and sham groups.

	Schizophrenia patients			Healthy controls		
	Verum	Sham	*p*-Value	Verum	Sham	*p*-Value
Age	33.2 (7.67)	39.9 (9.65)	0.54	30.8 (9.34)	32.6 (7.52)	0.64
Gender (m/f)	4/6	3/7	0.64	5/5	5/5	1.0
Handedness (r/l/a)	9/1/0	9/0/1	0.37	9/1/0	9/1/0	1.0
Education (years)	10.8 (1.93)	10.2 (1.14)	0.41	12.8 (0.42)	12.9 (0.32)	0.56
MWT-B	110.62 (20.6)	103.13 (9.99)	0.38	105.8 (14.48)	118.6 (20.81)	0.13
Duration of illness (years)	7.15 (5.87)	6.56 (5.22)	0.82	–	–	–
CDSS	5.9 (3.81)	4.5 (2.8)	0.36	–	–	–
GAF	56.9 (8.17)	62.67 (5.29)	0.09	–	–	–
CGI	4.2 (0.63)	3.7 (0.48)	0.06	–	–	–
PANSS score						
Positive	13.4 (4.22)	12.0 (3.86)	0.45	–	–	–
Negative	18.3 (3.89)	16.4 (6.19)	0.42	–	–	–
General	31.4 (5.74)	29.0 (8.82)	0.48	–	–	–
Total	63.1 (11.93)	57.4 (18.14)	0.42	–	–	–
CPZ equivalents	437.5 (244.73)	443.47 (490.26)	0.97	–	–	–
Antidepressants (y/n)	2/8	5/5	0.35	–	–	–
Mood stabilizer (y/n)	1/9	0/10	1.0			

*Data are presented as means ± SDs or frequencies*.

*MWT-B, German Multiple-Choice Vocabulary Test; CDSS, Calgary Depression Rating Scale for Schizophrenia; GAF, Global Assessment of Functioning Scale; CGI, Clinical Global Impression Scale; PANSS, Positive and Negative Syndrome Scale; f, female; m, male; r, right; l, left; a, ambidextrous; CPZ, chlorpromazine*.

*p-Values refer to a statistical comparison between verum and sham condition within the schizophrenia patient and healthy control group*.

This study was carried out in accordance with the recommendations of LMU Munich Medical Faculty ethics committee with written informed consent from all participants. All participants gave written informed consent in accordance with the Declaration of Helsinki. The protocol was approved by the LMU Munich Medical Faculty ethics committee. Participants were monetarily compensated for their participation.

The study was registered at https://www.drks.de (identifier: DRKS 00011665) and the WHO international clinical trials registry platform.^1^

### Study Protocol

**Figure 1:**
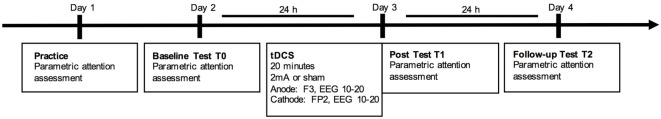
Flow-chart of the experiments.

The experiment consisted of four sessions taking place on consecutive days at about the same daytime each. On day 1, participants were trained on the respective tasks of the TVA-based assessment. On day 2, a baseline TVA-based assessment was conducted (T0) and participants were randomly assigned to either the verum or the sham tDCS condition. On day 3, the TVA-based assessment (T1) took place straightaway after the tDCS (anodal or sham), and on day 4, a follow-up assessment of the attentional parameters (T2) was conducted to examine for the consolidation of potential tDCS after-effects (see Figure 1).

### Attentional Assessment Based on Bundesen’s TVA

Primary outcome measure for attentional functioning was the parametric attentional assessment based on Bundesen’s TVA (15). Participants were tested at baseline, directly after, and 24 h after tDCS intervention (see Figure 1).

#### Framework of the TVA Approach

Theory of Visual Attention is a comprehensive mathematical model of selective attention (15, 54), which conceives of visual processing as a parallel competitive race of objects in the visual field for representation in a capacity-limited vSTM store (55): only those objects that are processed fastest will win the competition, that is, will be encoded in vSTM and thus become available for conscious report. The speed with which an object in the display is processed depends on the attentional weight assigned to it. Both bottom-up and top-down factors, such as, respectively, stimulus saliency and fit with instructed (selection-relevant) target features, are crucial determinants of the magnitude of the attentional weight allocated an object. Accordingly, only part of the objects will be represented within vSTM and can be used for further processing and goal-directed actions.

#### General Method for TVA Whole- and Partial-Report

Experiments took place in a dimly lit experimental laboratory at the Psychiatric Clinic of the Ludwig-Maximilians-Universität München (LMU Munich). TVA whole- and partial-report tasks were completed within one test session lasting about 1 h; task order was counterbalanced across participants. Stimuli were presented on a 27-inch PC monitor on a black background, with a refresh rate of 100 Hz and a resolution of 1,024 × 768 pixel. The viewing distance was set to approximately 60 cm. A trial started with the presentation of a white central fixation point (diameter: 1 cm) for 1,000 ms which participants were instructed to fixate throughout the whole trial. After 250 ms, red and/or blue letters were briefly flashed on the display with exposure durations that were adjusted individually according to a criterion value in a pretest. The letters were randomly selected from a predefined set (ABCDEFGHJKLMNOPRSTUVWXZ), with a letter never appearing repeatedly in one trial. The stimuli display was either followed by an empty black screen or a pattern mask consisting of a blue-red scattered square (≈1.5° visual angle) visible for 500 ms at each stimulus location. The participant was instructed to report the letters in any order and without speed stressing. The experimenter typed the responses on a keyboard and then initialized the next trial. After each block, a visual performance feedback informed the participants about the amount of correctly named letters out of all reported ones (in %). To avoid too conservative and too liberal responses, participants should aim for correctness between 70 and 90%.

##### TVA-Based Whole Report

**Figure 2:**
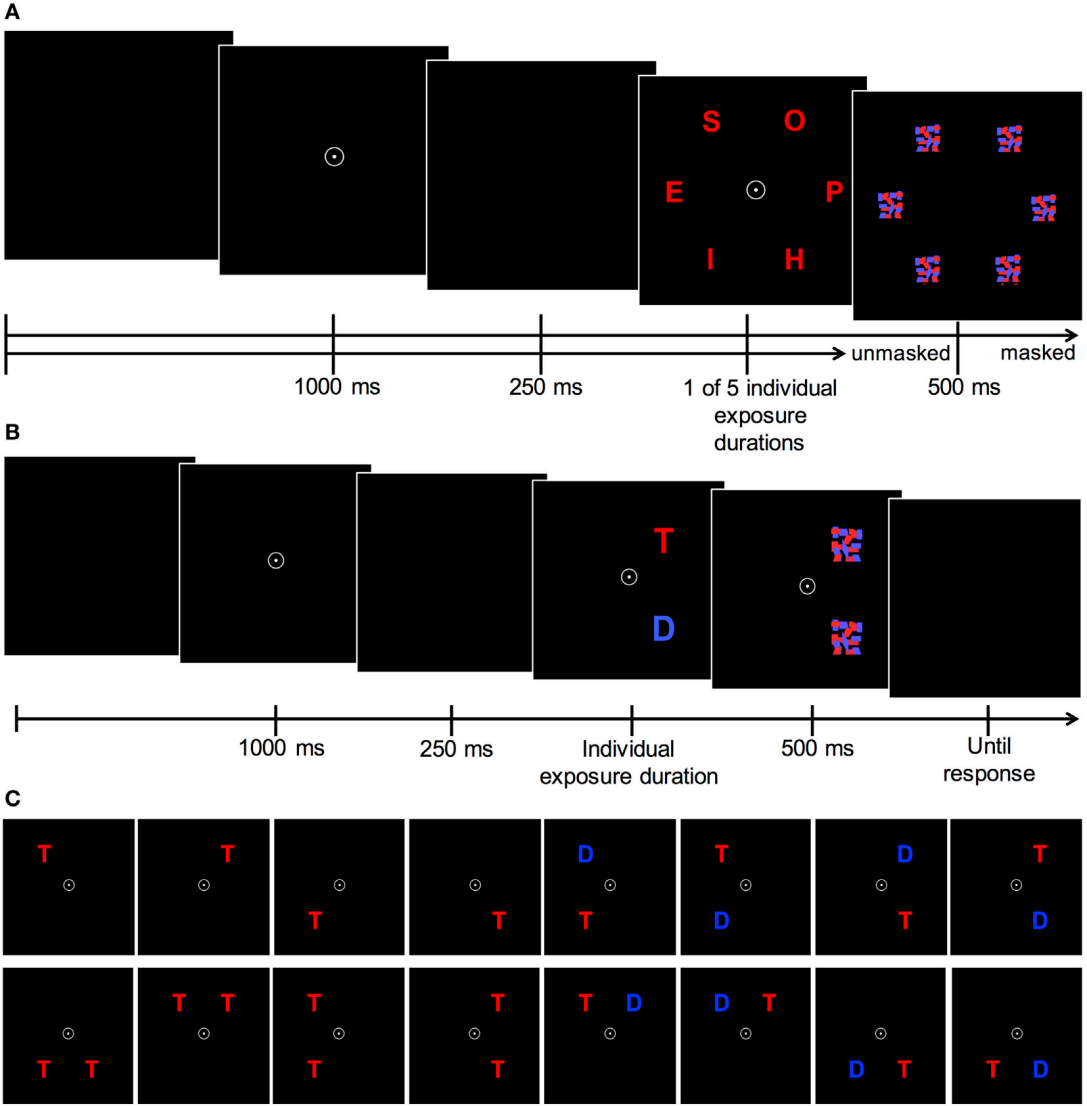
**(A)** Theory of Visual Attention (TVA) whole-report task procedure. After the presentation of a central fixation circle for 1,000 ms and a brief delay of 250 ms, six letters are flashed in an imaginary circle either in red or blue font for one of five individually adjusted exposure durations (identified in a pretest). In these five exposure duration conditions letters were masked for 500 ms. In two unmasked conditions, letters were presented in the second shortest and the longest exposure duration condition. **(B)** Trial sequence and **(C)** display types of TVA partial-report task. After the presentation of a central fixation for 1,000 ms and a brief delay of 250 ms, one of the 16 possible display types appears for a predetermined individual exposure duration. Following that, presented stimuli (T = target = red letters; D = distractor = blue letters) are masked for 500 ms. Adapted according to Gögler (18).

On each trial six letters, either all red or blue, appeared on an imaginary circle with a radius of 6 cm (5.73° of visual angle) around the fixation point (see Figure 2A). Participants had to identify and report as many letters as possible.

To find the five adequate exposure durations for a given participant a pretest of four blocks à 12 trials was conducted prior to the main whole-report task in each test session. Three types of trials were used in this pretest: two “easy” trials (i.e., one longer and one unmasked trial) and one adjusting trial in which initially, the six letters were flashed for 80 ms. If the participant could correctly identify at least one letter, exposure durations were decreased in steps of 10 ms until the lowest individual threshold, for which no letter could be reported anymore, was detected. This threshold was used to find an adequate set of four additional, longer exposure durations for the subsequent whole-report task (e.g., 10, 20, 40, 90, and 200 ms). In these five conditions, letters were masked. Additionally, in two unmasked conditions, letters were presented in the second shortest and the longest exposure duration condition. Consequently, there were seven “effective” exposure duration conditions. In unmasked trials an afterimage of the display emerges which extends the effective exposure durations by a constant duration which is defined by parameter μ (given in milliseconds) (56). The patient group’s average minimum exposure duration was 21 ms (SD = 4.47 ms) and did not differ significantly [*t*_(38)_ = −1.1, *p* = 0.32] from that of the control group, which was on average 20 ms (SD = 0 ms).

**Figure 3:**
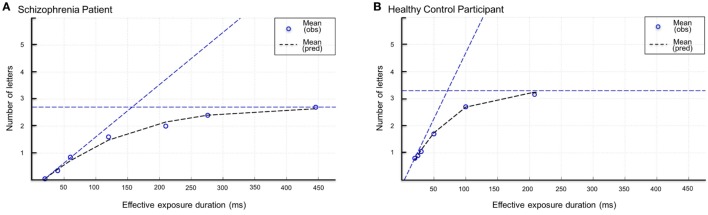
Whole-report performance of a representative schizophrenia patient **(A)** and a healthy control participant **(B)**. Mean number of correct letter reports as a function of exposure duration. Circles represent observed values (=obs), dashed lines represent the best fits of the observed scores by the applied model (pred = predicted). The estimate of visual short-term memory capacity *K* and processing speed *C* is indicated by the horizontal and diagonal dashed lines, respectively.

In total, the whole-report task consisted of 140 trials, separated into four blocks of 35 trials. Within each block, each display condition was presented equally often in randomized order. Based on the performance in the whole-report task, the individual processing capacity aspects reflected by the TVA parameters perceptual processing speed *C* and vSTM capacity *K*, can be estimated by mathematical data modeling (57). The probability of stimulus identification is modeled by an exponential growth function, relating the mean number of reported objects to the exposure duration. The use of seven effective exposure durations allows a broad depiction of the performance spectrum including early and late aspects of participant’s whole-report functions, and consequently a reliable model fit of the data. The growth parameter reveals the rate at which stimuli are processed (measured in visual elements per second; *C*), and the asymptote specifies the maximum number of objects that can be represented within vSTM store (*K*) (see Figure 3). Two further parameters, the threshold of conscious perception *t*_0_ and the effective *additional* exposure duration in unmasked displays μ, were also estimated (and did not differ significantly between groups and were not modulated by tDCS). These parameters merely serve the valid estimation of the parameters of interest but apart from this, they were of no further relevance in the present study.

##### TVA-Based Partial Report

On each trial either one or two letters (1 target, 2 targets or a target plus distractor) were flashed in the corners of an imaginary square (located 7.5 cm around the fixation point). If two letters were presented on the display, they either appeared in a row or in a column, but never diagonally. Participants had to report target letters (in red color) only, while ignoring distractors (blue). The stimulus arrays (see Figure 2B) appeared in randomized order and stimuli were always masked for 500 ms. The partial-report task consisted of 16 conditions (4 single-target T, 8 target plus distractor T-D, 4 dual-target conditions T-T), which were counterbalanced across all six blocks (see Figure 2C). A pretest (two blocks of 24 trials) was used to determine the individual exposure durations of the presented letter(s): first, letters were displayed with an initial exposure duration of 80 ms. If participants could identify two letters in the dual-target condition, exposure duration was decreased by steps of 10 ms until they could name, on average, one letter per trial correctly, whereas the exposure duration was increased by steps of 10 ms if they could not identify any letter. Exposure duration was kept unchanged, if they could identify one of the two target letters. Next, performance at the determined exposure duration was verified for the different experimental conditions in another turn of 24 trials. An adequate performance is denoted by correctly reported letters of 70–90% for single target conditions (T) and at least 50% for dual-target conditions. Otherwise exposure durations were in- or decreased manually by the experimenter and performance was rechecked in another turn of 24 trials. The patient group’s average exposure duration was 81.5 ms (SD = 32.85) and did not differ significantly [*t*_(38)_ = 0.95, *p* = 0.38] from those of the control group, that was on average 75.33 ms (SD = 27.69). The partial-report task consisted of 288 trials separated into six blocks of 48 trials. From the probability of stimulus identification, attentional weights are derived for targets (*w*_T_) and distractors (*w*_D_). Parameter α is defined as the ratio of distractor to target weights (*w*_D_/*w*_T_) and reflects top-down efficacy, i.e., the ability to prioritize task-relevant over task-irrelevant information. Values of α close to 0 indicate a high selectivity, i.e., targets receive more weight than distractors. Values of α close to 1 signify no selection and values larger than 1 imply that distractors receive more weight than targets, and hence were seen more easily.

### Transcranial Direct Current Stimulation

Transcranial direct current stimulation was delivered by a CE-certified stimulator (neuroConn, Germany) through saline-soaked surface sponge electrodes (35 cm^2^) at 2 mA for 20 min (plus 15 s fade-in and fade-out). The anode was placed above the left dlPFC located *via* F3 (EEG 10–20 system). This position covers Brodmann areas 8, 9, or 46 on the medial frontal gyrus—areas representative of the left dlPFC (58, 59). The cathode was placed above the right supraorbital area (FP2). This is the standard electrode montage used in physiological studies (60), and also in behavioral studies, this electrode montage was reported to modulate cognition both in healthy humans and patients (32, 38, 41).

Based on previous publications, sham stimulation was performed in the same way as verum stimulation, but the current was applied only for 30 s (plus 15 s fade-in and fade-out) (61, 62). Participants were randomly assigned to verum or sham tDCS by a computer-generated randomization list.^2^ To ensure double-blindness of both participants and experimenter, the experimenter did not have access to this list during the study; moreover, tDCS was performed by investigators not otherwise involved in the examination of patients. The study was designed as a parallel trial: 10 patients received verum left-anodal tDCS, and the remaining 10 patients underwent sham tDCS. Similarly, 10 healthy control participants received verum tDCS and 10 healthy controls received sham tDCS. During the stimulation, participants were not performing any task. This “offline” protocol was chosen as we were mainly interested in tDCS after-effects on attentional functions—both immediate and longer lasting ones of potential clinical relevance. Potential tDCS-induced adverse effects were examined by a *post hoc* comfort rating scale filled in by the participants (63).

### Data Analysis

Data were analyzed using IBM SPSS 22. The alpha level was set to 0.05. Baseline group differences in demographic and clinical variables were analyzed using independent *t*-tests for continuous variables and χ^2^ tests or, where appropriate, Fisher’s exact tests, for categorical variables. Baseline group differences in attentional performance as well as baseline differences in attentional performance, demographic, and clinical characteristics (patients) in participants assigned to the verum versus sham tDCS conditions within these two groups were analyzed by independent *t*-tests. Cohen’s *d* was calculated as a measure of the effect size for the group differences in attentional performance (64). To assess immediate and enduring effects of tDCS on the attentional parameters, two-way mixed ANOVAs were performed with time point (T0, T1, T2) as within-subject factor and stimulation condition (verum versus sham tDCS) as between-subjects factor, separately for the healthy control and the schizophrenia patient group. Mauchly’s test of sphericity was used to test the assumption of sphericity and, if significant, we applied Huynh–Feldt correction. In case of a significant interaction, the data were tested for simple main effects of time point, that is, we assessed differences in attentional parameters between time points for each level of the between-subjects factor stimulation condition.

By means of χ^2^ tests, we assessed whether the number of participants who believed to have received verum stimulation differed between the verum and sham conditions. Furthermore, comfort ratings were compared between participants of the verum and sham conditions through independent *t*-tests.

## Results

All schizophrenia patients and healthy control participants completed the entire experiment. No unexpected adverse effects of tDCS, such as skin burns, pain, or headache, were reported or revealed by the comfort rating questionnaire.

### Demographic and Clinical Characteristics

The schizophrenia patient and healthy control groups were matched according to age (*p* = 0.09), gender (*p* = 0.34), IQ (*p* = 0.37), and handedness (*p* = 0.51). The two groups differed significantly with respect to education level (*p* < 0.01). In both groups, participants receiving verum and sham stimulation did not differ significantly with respect to any of the demographic and clinical characteristics (all *p*s ≥ 0.06; Table 2).

### Baseline Task Performance—Healthy Control versus Schizophrenia Patient Group

#### Whole-Report Results

In Figure 3, the mean number of correct reports as a function of the (effective) exposure duration is depicted for one representative schizophrenia patient and one healthy control participant. The curves represent the maximum likelihood fits to the observed data, which correlated fairly well. TVA’s best fits accounted for *r*^2^ = 92% of the variance of the observed mean scores at the different exposure durations. Based on mathematical data modeling of the performance (correct letter reports) in the whole-report task (57), individual estimates were derived for perceptual processing speed *C* and vSTM storage capacity *K*. Table 3 depicts all means and SDs of the respective baseline TVA parameters in the healthy controls and schizophrenia patients.

**Table 3:** Theory of Visual Attention whole- and partial-report parameters at baseline for the schizophrenia patient and healthy control group.

	Schizophrenia patients		Healthy Controls		*p*-Value
	M	SD	M	SD	
*C*	29.55	21.21	43.86	19.18	0.03
*K*	3.01	0.78	3.67	0.94	0.02
α	0.35	0.18	0.36	0.22	0.93

*C, visual perceptual processing speed (elements/s); K, visual short-term memory capacity (number of elements); α, efficiency of top-down control. p-Values refer to a statistical comparison between the schizophrenia patient and healthy control group*.

##### Perceptual Processing Speed *C*

**Figure 4:**
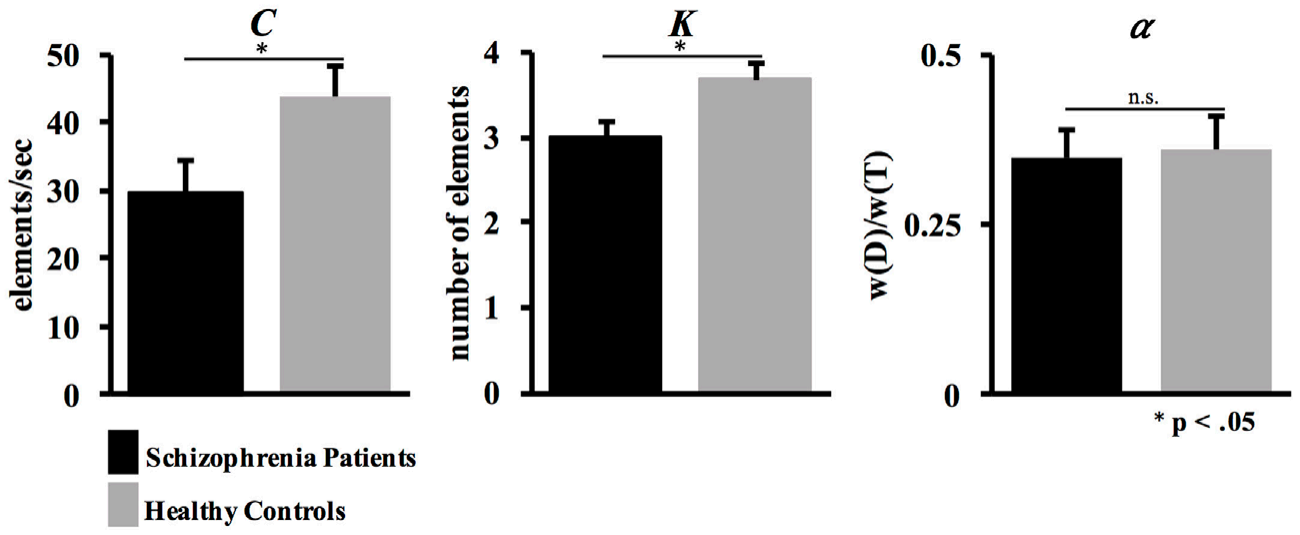
Whole- and partial-report results. Mean estimates and SEs for the Theory of Visual Attention parameters processing speed *C*, short-term memory capacity *K*, and efficiency of top-down control α.

Analysis revealed processing speed to be significantly lower in schizophrenia patients (M = 29.55 items/s, SD = 21.22) than in healthy controls [M = 43.86 items/s, SD = 19.18; *t*_(38)_ = 2.24, *p* = 0.03] (see Figure 4). This effect is also illustrated by the slope of the whole-report functions depicted in Figure 3, which is steeper for the representative control participant than for the schizophrenia patient. Thus, the rate of visual information uptake within a given unit of time is significantly reduced in schizophrenia. Computation of Cohen’s *d* yielded a medium to large effect size (*d* = 0.7) and a 43% non-overlap of the two distributions of *C* scores.

##### vSTM Capacity *K*

Analysis disclosed vSTM storage capacity to be significantly decreased in schizophrenia patients (M = 3.01, SD = 0.78 items) compared to healthy controls [M = 3.67, SD = 0.94 items; *t*_(38)_ = 2. 42, *p* = 0.02] (see Figure 4). As can be seen from Figure 3, as exposure duration increases, report performance approaches an asymptotic level, which represents the (depicted individuals’) vSTM storage capacity: the patient’s asymptote is lower than that of the healthy control participant—illustrating that the mean number of items that can be represented in vSTM is reduced in schizophrenia. The effect size is large (*d* = 0.8), with a 47.4% non-overlap of the two distributions of *K* scores.

#### Partial-Report Results

Mathematical modeling of performance in the partial-report task permits inferences to be drawn about the functioning of attentional selectivity, reflected in the top-down control parameter α (57). There was again a close correspondence between the observed performance at the different exposure durations and TVA’s best fits to the data: the predicted values accounted for *r*^2^ = 91% of the variance of the observed mean scores.

##### Top-Down Control α

Analysis revealed statistically comparable estimates of top-down control α between schizophrenia patients (M = 0.35, SD = 0.18) and healthy controls [M = 0.36, SD = 0.22; *t*_(38)_ = 0.09, *p* = 0.93] (see Figure 4).

### Immediate and Enduring Effects of tDCS on Attentional Parameters

#### Healthy Controls

**Figure 5:**
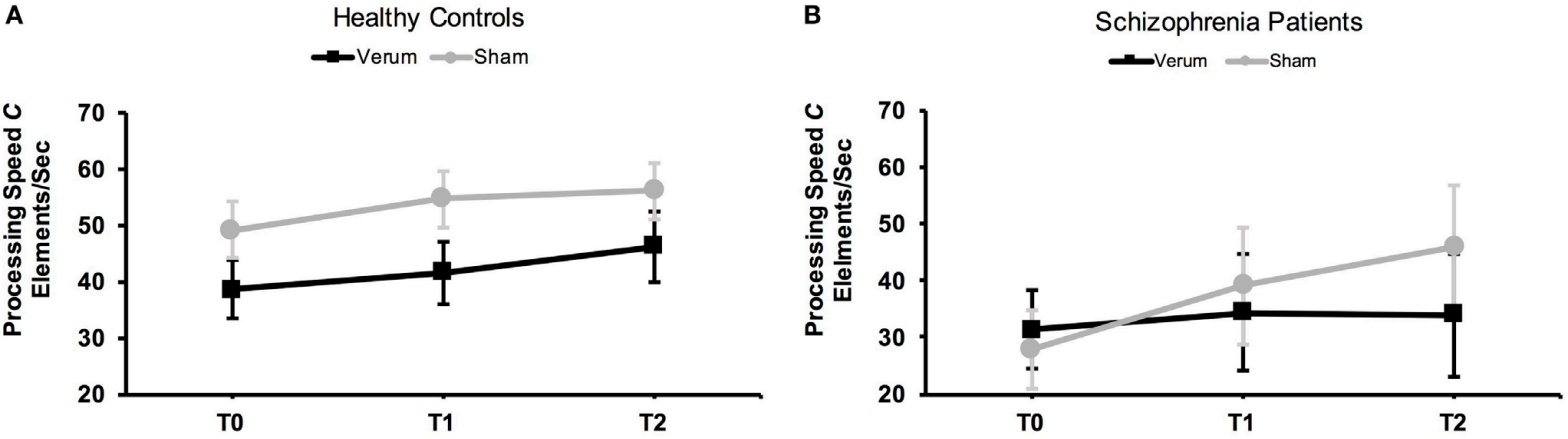
Effects of transcranial direct current stimulation (tDCS) on mean perceptual processing speed *C* in healthy controls **(A)** and schizophrenia patients **(B)**. Processing speed was assessed at baseline (T0), directly after tDCS (T1), and 24 h after tDCS (T2). Error bars represent SEMs.

For processing speed *C*, the ANOVA revealed the main effect of time point to be significant: processing speed increased from baseline to post and then to follow-up test [*F*_(2, 36)_ = 4.05, *p* = 0.03; see Figure 5]. No other significant effects were obtained (all *p*s ≥ 0.12; for means and SDs, see Table 4). As these are the results of our in-house, “historical healthy-control cohort,” we refer to Ref. (18) for a more detailed description of the findings.

**Table 4:** Theory of Visual Attention whole- and partial-report parameters in the healthy control and schizophrenia patient groups for the three time points (T0, T1, T2).

tDCS condition		T0		T1		T2	
		M	SD	M	SD	M	SD
**Healthy controls**							

*C*	Verum	38.59	16.76	41.53	17.11	46.18	19.92
	Sham	49.13	20.84	54.69	22.49	56.09	28.29
*K*	Verum	3.47	0.82	3.33	0.84	3.48	0.78
	Sham	3.88	1.04	3.86	0.93	4.01	1.02
α	Verum	0.27	0.20	0.22	0.26	0.33	0.16
	Sham	0.45	0.21	0.42	0.20	0.39	0.11

**Schizophrenia patients**							

*C*	Verum	31.36	16.83	34.33	19.02	33.92	19.01
	Sham	27.73	25.69	39.02	41.92	46.01	44.91
*K*	Verum	3.40	0.64	3.47	0.59	3.64	0.64
	Sham	2.63	0.75	3.06	0.69	3.03	0.59
α	Verum	0.36	0.15	0.40	0.17	0.42	0.27
	Sham	0.34	0.21	0.38	0.18	0.36	0.13

*tDCS, transcranial direct current stimulation; *C*, visual perceptual processing speed (elements/s); *K*, visual short-term memory capacity (number of elements); α, efficiency of top-down control*.

#### Schizophrenia Patients

##### Baseline Comparisons between Verum and Sham Condition

For the schizophrenia patients, analyses revealed no significant baseline differences between the verum and sham tDCS conditions for the TVA parameters processing speed *C* and top-down control α (all *t*s ≤ 0.37, all *p*s ≥ 0.30). However, there was a significant difference with respect to parameter *K*: patients in the verum condition exhibited a significantly higher vSTM capacity (M = 3.39, SD = 0.64 items) than patients in the sham condition [M = 2.63, SD = 0.75 items; *t*_(18)_ = 2.44, *p* = 0.03]. See Table 4 for respective means and SDs.

##### tDCS Effects on Whole-Report Performance

For processing speed, analysis revealed a significant main effect of time point [*F*_(2, 36)_ = 6.72, *p* = 0.01] and a significant interaction between tDCS condition and time point [*F*_(1.64, 29.44)_ = 3.67, *p* = 0.04] in the patient group. Separate ANOVAs computed for the two tDCS conditions (to follow up the interaction) revealed the effect of time point to be significant for the sham group [*F*_(1.41, 12.67)_ = 6.48, *p* = 0.02]: processing speed *C* increased somewhat from the baseline (M = 27.73, SD = 25.69 items/s) to the post test (M = 39.02, SD = 41.92 items/s), yielding a trend-level difference [*t*_(9)_ = −2.04, *p* = 0.07]; and there was a further increase to the follow-up test, manifesting in a statistically reliable difference between the baseline and follow-up tests [M = 46.01, SD = 44.91 items/s; *t*_(9)_ = −2.87, *p* = 0.02] (see Figure 5). On average, patients receiving sham stimulation could process some 18 elements/s (67%) more at the follow-up compared to the baseline test. A Cohen’s *d* of 0.50 indicated a medium effect size. In contrast to the sham group, there was no main effect of time point for the verum group [*F*_(2, 18)_ = 0.69, *p* = 0.51], that is, processing speed *C* remained stable across the various time points of testing. At the single-subject level, only a single patient (out of 10) in the verum condition showed an increase in the parameter processing speed *C* from baseline to follow-up testing considering a threshold of ≥50% improvement. In contrast, 7 out of 10 patients in the sham condition showed an increase in processing speed (≥50%) from baseline to follow-up testing. A Fisher’s exact test between tDCS condition (sham/verum) and “improvement ≥50%” (yes/no) yielded a significant association between tDCS condition and “improvement,” *p* = 0.02.

For the parameter vSTM storage capacity *K*, analysis yielded a significant main effect of time point [*F*_(2, 36)_ = 4.87, *p* = 0.01]: the patients’ ability to represent items in vSTM increased from baseline to post and further to follow-up test. However, the time point × tDCS condition interaction was not significant [*F*_(2, 36)_ = 1.36, *p* = 0.27].

##### tDCS Effects on Partial-Report Performance

For the parameter top-down control α, analysis yielded no statistically reliable effects (all *p*s ≥ 0.56).

We repeated these analyses using “GAF” and “CGI” as covariates, which confirmed the results for all three parameters and, therefore, indicate that the observed tDCS effect in the schizophrenia patient group cannot be explained by differences in these clinical characteristics between the verum and sham condition.

### Integrity of Blinding and Comfort Rating

Participants were successfully blinded: of the schizophrenia patients, 9 patients in the verum and 7 in the sham condition indicated that they had received verum stimulation [χ^2^(1) = 1.25, *p* = 0.26]. Of the healthy controls, seven participants in the verum and three in the sham condition believed that they had received verum stimulation [χ^2^(1) = 3.20, *p* = 0.07]. Within both the schizophrenia patient and the healthy control group, there were no significant differences between participants in the verum and sham conditions with respect to comfort ratings (sum score of the 10-point Likert scales) relating to the time during and after the stimulation (all *t*s ≤ 2.01, all *p*s ≥ 0.06).

## Discussion

The present study had two objectives. First, we applied mathematical data modeling based on Bundesen’s TVA to isolate the particular attentional deficits in schizophrenia patients compared to healthy controls. Second, we assessed whether these deficits could be modulated by means of a single, 20-min tDCS session with 2 mA over the dlPFC. In brief, we found an altered pattern of attentional parameters, expressed by significantly reduced visual processing speed *C* and vSTM storage capacity *K*. However, contrary to our hypothesis, we did not find evidence that verum tDCS, compared to sham stimulation, would improve attentional functioning. Instead, a differential development from baseline to follow-up assessment indicated that the normal, practice-dependent increase in visual processing speed that occurs with repeated application of the whole-report task (shown by healthy controls and patients in the sham group) disappears when verum tDCS is applied to the left dlPFC in schizophrenia patients.

### Visual Perceptual Slowing and vSTM Capacity Deficit at Baseline Assessment

To our knowledge, this is the first study applying TVA-based parametric attentional assessment in schizophrenia patients. This enabled us to isolate an impairment of general attentional capacity (without an impairment of attentional selectivity) as the primary factor compromising visual attentional functioning in schizophrenia. Specifically, at baseline, schizophrenia patients exhibited significantly reduced visual processing speed *C* and vSTM storage capacity *K*. The neural interpretation of the TVA (NTVA) (54) attributes processing speed changes to changes in either the activation level or the overall number of the neurons that are devoted to processing the visual information presented. On this notion, our results imply that schizophrenia leads to a reduced overall arousal level of the brain, likely owing to changes in the excitability of the alertness network. NTVA furthermore assumes that vSTM storage relies on a cortical–thalamic circuitry supporting activity in reverberating loops. Accordingly, our finding of schizophrenia patients exhibiting a reduction in the amount of information they can maintain in vSTM would imply that the functional integrity of this system is impaired.

From a general point of view, our findings are in line with previous reports of processing speed and vSTM deficits in schizophrenia revealed by means of various other testing procedures (6, 65–67). They also replicate high effect sizes for differences in vSTM storage capacity estimates between schizophrenia patients and healthy controls based on experimental measures (5, 10, 68). However, using the TVA approach, which is based on a well-grounded computational theory, we could assess relevant and distinct attentional components of interest in an independent manner—without confounding speed of information uptake, vSTM capacity, and distractibility (44, 69, 70). Extracting these components within the same tasks with identical stimuli and response requirements revealed an attentional profile specific for schizophrenia. As selectivity aspects of attention were not significantly altered in schizophrenia patients compared to healthy controls, we can rule out that the capacity limitations are secondary consequences of impaired top-down control. This is again in line with previous reports of preserved attentional control of information encoding into short-term memory (11). Note that the present results have no bearing with regard to top-down controlled processing in situations with (bottom-up) highly salient distractors. There is evidence that patients with schizophrenia exhibit deficits in attentional selection when salient distractors compete for attentional selection (12). Furthermore, our results are unlikely attributable to unspecific antipsychotic drug-induced motor side-effects, as the TVA-based assessment requires only unspeeded verbal responses. Similarly, these visual attentional deficits are unlikely attributable to eye movement impairments, often reported in schizophrenia patients [e.g., Ref. (71)], as the TVA-based assessment uses very brief exposure durations below the latency of saccadic eye movements. Besides, eye movement abnormalities should be reflected in elevated perceptual thresholds (parameter *t*_0_). However, this parameter was found to be not significantly different between patients and healthy controls. The latter also implies that motivational impairments unlikely underlie the observed visual attentional deficits.

### tDCS-Based Modulation of Attentional Parameters

Unexpectedly, we found a significant increase in the (impaired) parameter processing speed *C* at the follow-up assessment only in patients receiving sham (but not verum) tDCS. That is, single-session verum tDCS over the dlPFC appears to be ineffective, or maybe even harmful, for improving attentional functioning in schizophrenia—a finding that echoes those of a recent study (72) which assessed the effect of 2-week dlPFC tDCS on the secondary outcomes WM (SOPT), processing speed (TMT-A), and executive functioning (TMT-B) in schizophrenia patients with predominantly negative symptoms. In contrast, in the present study, tDCS did not influence information uptake processes in healthy control participants. This differential effect of tDCS on the processing speed parameter *C* in healthy participants and in those suffering from schizophrenia may be explained by unexpected effects of tDCS in schizophrenia. Schizophrenia is a disorder of disturbed neuronal plasticity with alterations in glutamatergic neurotransmission (73), is characterized by a dysfunction in interneurons and GABAergic neurotransmission affecting microcircuity (74) and a dopaminergic dysbalance is evident (75). tDCS effects are dependent on NMDA, GABA, and dopaminergic receptor activity (76) and have been discussed not only to act at the soma of pyramidal neurons, but possibly also on the interneuron level (77). Due to these alterations that are all related to the mode of action of tDCS, one could speculate that tDCS may have unexpected clinical and neurophysiological effects in schizophrenia patients.

Two potential mechanisms, which cannot be differentiated based on our study, might be responsible for the reduction in processing speed increase from baseline to follow-up testing. First, given that we observed practice-dependent enhancement of visual processing speed from baseline to follow-up assessment in healthy participants in both the sham and the verum group and in schizophrenia patients in the sham group, the application of tDCS in schizophrenia patients might interfere with practice effects that likely rely on implicit procedural learning of performing the whole-report task. Alternatively, tDCS might impact processing speed by reducing the overall arousal level in schizophrenia patients’ brains for at least 24 h. Thus, for patients in the verum group, even though they received the same amount of whole-report training as the sham group, the training benefits are effectively nulled by the lowered arousal level. The present results highlight the need for further safety assessments in tDCS studies involving psychiatric patients and, more particularly, for more systematic evaluation of tDCS effects on cognition before embarking on large-scale clinical trials.

Our results suggest that the applied stimulation parameters—tDCS for 20 min at 2 mA over the left dlPFC—are not appropriate for ameliorating attentional dysfunctions (as assessed by TVA) in schizophrenia patients. This appears to be at odds with other studies that used similar tDCS protocols and reported beneficial effects in reducing negative symptoms and improving cognitive functions in schizophrenia (38, 72) and other psychiatric disorders (32). Reasons for the unfavorable effects on cognition obtained in the present study might be the relatively high intensity and duration of the stimulation. Although these settings are typical for the field of cognitive neuroscience, they have yielded unexpected effects in previous tDCS studies of motor cortex, where non-linear effects of dosage have been reported with healthy participants: greater tDCS intensity, rather than being associated with higher efficacy of stimulation, shifted the excitability alterations (78). Moreover, the individual response variability of tDCS at both 1 and 2 mA (79–81) may hamper the efficacy of our intervention in the given population offering an alternative explanation of the here reported unexpected findings. As the positioning of the electrodes can impact tDCS effects (82), our negative finding might also have been the result of non-optimal electrode montage: it cannot be ruled out that the “reference” electrode over the right supraorbital area induced confounding effects and that, for instance, larger (being less active) or extracephalic reference electrodes might have produced a different outcome. Likewise, although in imaging studies this kind of electrode configuration was shown to modulate fronto-parietal attention networks (41), the position of the “active” electrode above the left dlPFC might have been inappropriate for modulating visual attentional functions in schizophrenia patients. Finally, it should be borne in mind that schizophrenia patients exhibit significant alterations in dopaminergic transmission and that all antipsychotics act on dopamine receptors. In this context, dopaminergic modulation has been shown to impact the efficacy of tDCS in a non-linear manner, resulting, for example, in a reversal of plasticity effects (83, 84).

### Limitations

First, the sample size of this proof-of-concept study, while being comparable with other studies in the field, was relatively small, increasing the probability of a type II error. Therefore, findings must be confirmed in a larger sample before generalizing these results. The limited sample size and the use of a between-subjects design may limit our findings. Albeit not likely, as the patients in the verum and sham conditions were comparable with respect to the initial visual processing speed parameter, it cannot be excluded that the observed tDCS effect may be explained in part by differences in sociodemographic characteristics between both groups. Moreover, as all patients received antipsychotic medication, cognitive parameters could not be investigated independently of potential confounding medication effects. However, Pearson correlations between CPZ and cognitive performance (*C, K*, α) at study inclusion did not correlate significantly (*C*: *r* = 0.37, *p* = 0.11; *K*: *r* = 0.44, *p* = 0.18; α: *r* = 0.41, *p* = 0.08), indicating that antipsychotic doses had no impact on our outcome variables. Regarding tDCS effects, we cannot rule out that these may have resulted from interactions between medication and tDCS yielding the unfavorable outcome. As outlined above, antipsychotic drug-induced dopaminergic modulations can affect tDCS-induced changes in cortical excitability and plasticity (83, 84). However, as tDCS is considered an add-on treatment option, experimental trials with medicated patients would, arguably, be representative for a clinical setting.

### Conclusion

In the present study, employing TVA-based parametric assessment of attentional functions, schizophrenia patients were revealed to exhibit a characteristic pattern of attentional capacity impairments: a significantly reduced rate of visual information uptake (per time unit) and a significantly reduced vSTM storage capacity (in terms of the number of items that can be maintained simultaneously). Combining this approach with a tDCS intervention revealed that 20 min of 2 mA prefrontal tDCS interferes with (rather than enhances) practice effects on visual processing speed in schizophrenia. This finding of a potential tDCS-induced disrupting effect on the here investigated cognitive domain calls for further investigation and highlights the need for more neuroscience-based research in schizophrenia.

## Ethics Statement

This study was carried out in accordance with the recommendations of LMU Munich Medical Faculty ethics committee with written informed consent from all participants. All participants gave written informed consent in accordance with the Declaration of Helsinki. The protocol was approved by the LMU Munich Medical Faculty ethics committee.

## Author Contributions

JF, KF, HM, and AH designed the study. NG conducted the behavioral assessment and analyzed the data. IP was responsible for the clinical ratings. TO-S, NF, and WS applied the tDCS. AH and KF supervised the project. NG, KF, and AH wrote the manuscript. All authors contributed to manuscript revision.

## Conflict of Interest Statement

The authors declare that no financial support or compensation, apart from the above-mentioned grants and income received from primary employers, has been received. However, other conflicts of interests not related to this publication are: AH has received speaker funding through Desitin, Otsuka and Lundbeck. He was a member of a Roche and Janssen-Cilag Advisory Board; WS has received speaker funding through Mag and More. All other authors report no biomedical financial interests or potential conflict of interests.

## Footnotes

^1^http://apps.who.int/trialsearch/Trial2.aspx?TrialID=DRKS00011665.

^2^https://www.random.org/lists/.
